# Seabird Tissue Archival and Monitoring Project (STAMP) Data from 1999-2010

**DOI:** 10.6028/jres.126.028

**Published:** 2021-12-17

**Authors:** Nathan A. Mahynski, Jared M. Ragland, Stacy S. Schuur, Rebecca Pugh, Vincent K. Shen

**Affiliations:** 1 National Institute of Standards and Technology, Gaithersburg, MD, 20899 USA; 2 National Institute of Standards and Technology, Charleston, SC 29412 USA

**Keywords:** biorepository, dataset, environmental chemistry, marine

**Data DOI:** https://doi.org/10.18434/mds2-2431

## 1 Summary

The multi-entity, long-term Seabird Tissue Archival and Monitoring Project (STAMP) [1] has collected eggs from various avian species throughout the North Pacific Ocean for over 20 years to create a geospatial and temporal record of environmental conditions. Over 2,500 samples are currently archived at the NIST Biorepository [2] at Hollings Marine Laboratory [3] in Charleston, South Carolina. Longitudinal monitoring efforts of this nature provide invaluable data for assessment of both wildlife and human exposures as these species often consume prey (*e.g.*, fish) similar to, and from sources (*e.g.*, oceanic) comparable to, human populations nearby. In some areas, seabird eggs also comprise a significant part of subsistence diets providing nutrition for indigenous peoples. Chemometric profiles and related health implications are known to differ across species [4]. Eggs, however, can be difficult to assign to a species unless the bird is observed on the nest from which the sample was collected due to similar appearance within a genus and sympatric nesting behavior. This represents a large point of uncertainty for both wildlife managers and exposure researchers alike.

Here we have curated analytical data for eggs collected from 1999 to 2010 on a subset of species and analytes that were measured regularly and reasonably systematically. Included in this publication are 487 samples analyzed for 174 ubiquitous environmental contaminants such as brominated diphenyl ethers (BDEs), mercury, organochlorine pesticides, and polychlorinated biphenyls (PCBs). Data were collated to form a dataset useful in chemometric and related analyses of the marine ecosystem in the North Pacific Ocean. Here we describe the data processing performed and other characteristics of the dataset.

## 2 Data Specifications

**Table:** 

**NIST Operating Unit**	Material Measurement Laboratory
**Format**	Comma separated values
**Instrument**	Cryogenic and fresh homogenization, pressurized fluid extraction (PFE), size exclusion chromatography (SEC), solid phase extraction (SPE), gas chromatography (GC), mass spectrometry (MS), inductively coupled plasma MS (ICP-MS), liquid chromatography (LC)/MS/MS, neutron activation, continuous-flow isotope ratio mass spectrometry (CFIRMS).
**Spatial or Temporal Elements**	North Pacific Ocean, 1999-2010
**Data Dictionary**	https://doi.org/10.18434/mds2-2431
**Accessibility**	All datasets submitted to *Journal of Research of NIST* are publicly available.
**License**	https://www.nist.gov/director/licensing

## 3 Methods

### 3.1 Data Measurement and Collection

Seabird eggs were collected, processed, and archived using standardized protocols; a cryohomogenization process was used until 2005 for murre eggs to sub-sample the egg contents into multiple, homogeneous aliquots. A fresh homogenization was used for gull eggs in 2005 as well as subsequent samples for all species of STAMP [5–7]. Sample characteristics were recorded for all samples entering the NIST Biorepository. Each sample and associated aliquots were assigned persistent identifiers (PID) minted by the biorepository database (Freezerworks^1^1Any mention of commercial products is for information only; it does not imply recommendation or endorsement by NIST.) [8]. Those characteristics (species, location, morphology, provenance, etc.) were then linked to analytical result data using the aliquot PID to serve as metadata for more sophisticated mathematical analyses. Eggs were analyzed as previously described in Refs. [9–11]. Methods by batch are described in the appendix [12] which we briefly summarize here.

• Brominated Diphenyl Ethers (BDEs), Organochlorine Pesticides, Polychlorinated Biphenyls (PCBs): Samples were extracted with pressurized fluid extraction (PFE) using sodium sulfate (1999 eggs) or diatomaceous earth, which were cleaned using size exclusion chromatography (SEC) followed by semi-prep aminopropylsilane fractionation (1999–2000 eggs) or solid phase extraction (SPE) with alumina and analyzed by gas chromatography (GC) with electron capture detection (ECD; 1999–2000 eggs) or GC-mass spectrometry (MS). See the appendix [12] for more details. SRM 1946 Lake Superior Fish Tissue and, after 2001, an in-house murre egg control material, were used for quality assurance.
• Butyltins: Mono- (MBT), Di- (DBT) and Tributyltin (TBT) were analyzed by digestion with tetramethylammonium hydroxide solution in open focus microwave, adjusting pH to 5, derivatizing with tetraethylborate in hexane, cleaned with alumina solid phase extraction (SPE) and analyzed by speciated isotope dilution and gas chromatography inductively coupled plasma mass spectrometry (SIDGC/ICP-MS). CRM CE477 Mussel Tissue and SRM 1974b Organics in Mussel Tissue were used for quality assurance.
• Carbon and Nitrogen Isotopes: Samples were shipped in nitrogen vapor freezers to Keith Hobson at Environment Canada’s Stable Isotope Hydrology and Ecology Research Laboratory (SIHERL) where they were first freeze-dried, then lipids were extracted using a 2:1 chloroform:methanol soak; filtrates were dried before powdering and subsampling them for analysis. Carbon and nitrogen stable isotope ratios were obtained by combustion using continuous-flow isotope ratio mass spectrometry (CFIRMS). The ratios were expressed in delta (*δ*) notation relative to the Vienna PeeDee Belemnite (VPDB) or AIR standards for carbon and nitrogen, respectively. The albumen standard, manufactured in-house at the SIHERL facility, has been calibrated to International Atomic Energy Agency (IAEA) Vienna PDB and Atmospheric Air standards for *δ* 13C for *δ* 15N, respectively. Quality assurance was performed using an SIHERL in-house albumen standard, calibrated to International Atomic Energy Agency (IAEA) Vienna PDB and Atmospheric Air standards for *δ* 13C for *δ* 15N, respectively.
• Mercury (Hg): Samples were isotopically spiked with ^201^Hg calibrated with SRM 3133 Mercury Standard Solution, digested with nitric acid (and 2008–2010 eggs with hydrogen peroxide) in a microwave (closed cell in 1999–2001, 2008 eggs and open focus in 2002–2005, 2009 eggs) and analyzed by cold vapor-isotope dilution-inductively coupled plasma mass spectrometry (CV-ID-ICPMS). Beginning in 2010, Hg was determined by direct mercury analyzer with external calibration utilizing SRM 3133 Mercury Standard Solution. SRM 2976 Mussel Tissue (1999–2001), SRM 1947 Lake Michigan Fish Tissue (2002–2005 eggs) and an in-house murre egg control material were used for quality assurance (2002 and later eggs).
• Mercury Isotopes: Samples were digested in nitric acid in open focus microwave and analyzed by ICP-MS using NIST SRM 3133 Mercury Spectrometric Solution as calibration.
• Per- and polyfluorinated alkyl substances (PFAS): Samples were double extracted using potassium hydroxide (KOH) in methanol, the supernatant evaporated with nitrogen gas, and cleaned with solid phase extraction (SPE) using ENVI-Carb cartridges and methanol; samples were reduced in volume with nitrogen gas, filtered and analyzed using liquid chromatography/tandem mass spectrometry (LC/MS/MS) using Betasil C8 and Luna PFP columns.
• Trace elements: A subset of eggs collected in 1999 (common murre eggs 10-19 from St. Lazaria) were irradiated in pneumatic tubes for 2 hours and then allowed to decay for each element (except arsenic) one to two months to decrease activity from shorter-lived isotopes. Gamma radiation was collected for 9 hours to 2 days using the “Max” detector and associated electronics consisting of a high voltage supply, a spectroscopy amplifier, and an analog-to-digital converter (ADC). For arsenic, decay was 5 days and gamma radiation was collected for 2 hours to 4 hours using the “Linda” detector and associated electronics consisting of a high voltage supply, a spectroscopy amplifier, and an ADC. SRM 1566a Oyster Tissue and SRM 1575a Pine Needles were used as control materials.
Trace elements were nitric acid and hydrogen peroxide-assisted microwave digested and analyzed by inductively coupled plasma mass spectrometry (ICP-MS) with a standard low-volume glass impact bead spray chamber (Peltier cooled at +3 *^◦^*C), and concentric glass nebulizer in collision cell mode. SRM 1947 Lake Michigan Fish Tissue (1999–2005 eggs), in-house murre egg control material, QC04-ERM1 (1999–2005 eggs), and SRMs 1566b Oyster Tissue and 1577c Bovine Liver (2012–2014 eggs) were used for quality assurance.

### 3.2 Data Processing

**Fig. 1:**
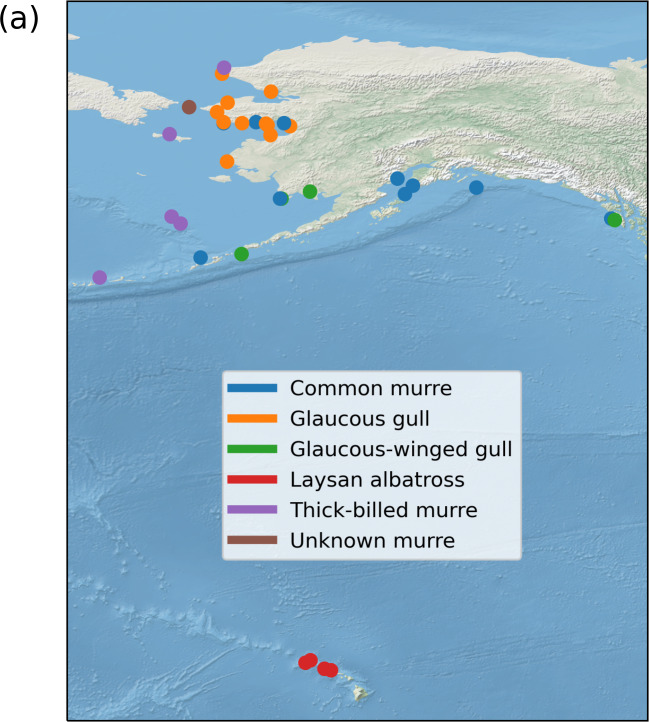

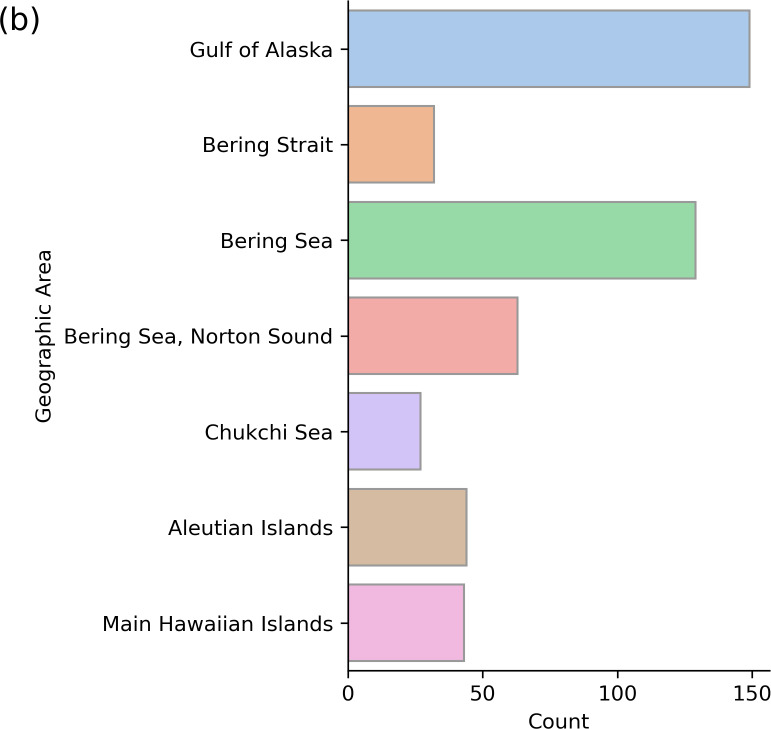

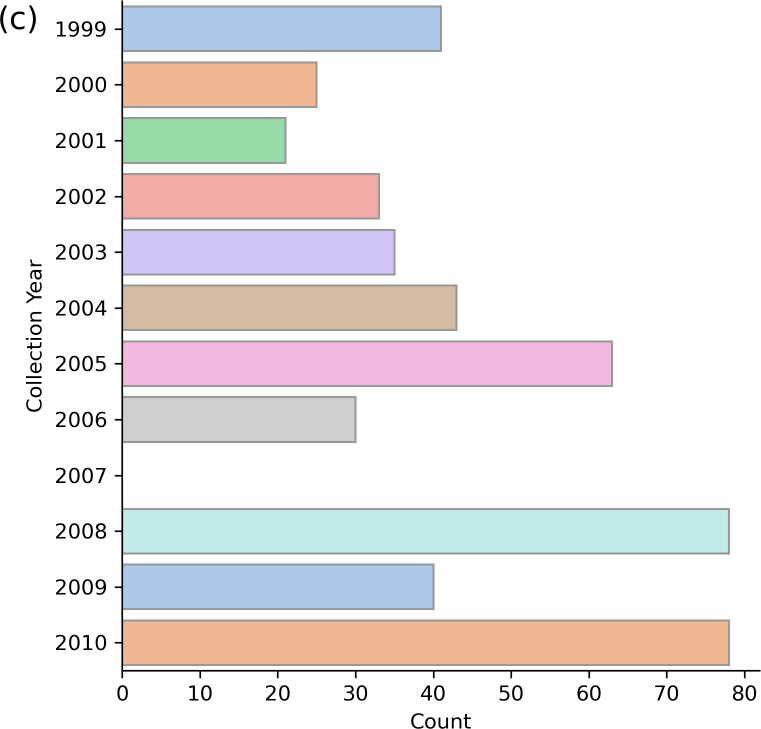

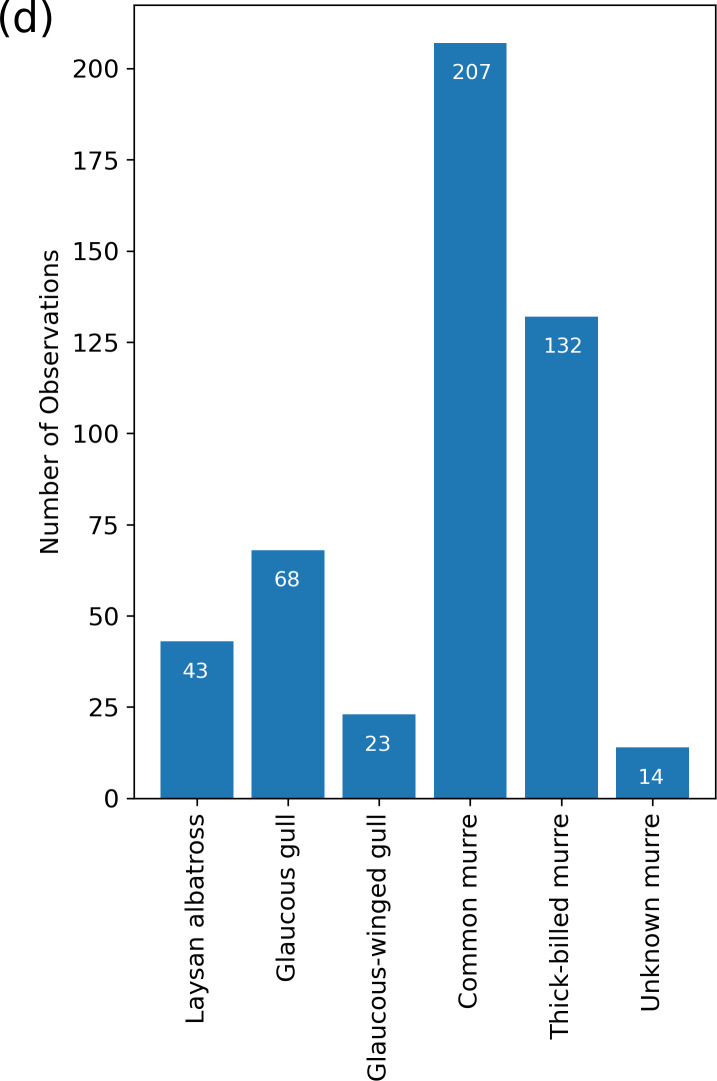
A summary of the raw data taken from the NIST Biorepository for further processing. (a) Location of the colonies selected, colored by the majority class found there. There may be multiple species found at a given colony. This plot was produced using the cartopy package [14]. (b) Breakdown of sample origins based on broad geographic area. (c) A breakdown based on the year the samples were collected. (d) A breakdown of the samples based on species.

The NIST Biorepository represents a collection point for many different studies investigating a wide range of topics, and is therefore heterogeneous and sparse by nature. We identified a subset of the biorepository spanning five species of seabirds including: Common murre (*Uria aalge*, Pontoppidan 1763), Thick-billed murre (*Uria lomvia*, Linnaeus 1758), Glaucous gull (*Larus hyperboreus*, Gunnerus 1767), Glaucous-winged gull (*Larus glaucescens*, Naumann 1840), and Laysan albatross (*Phoebastria immutabilis*, Rothschild 1893). The geographic distribution of different colonies, labeled according to the majority class is given in Fig. 1(a). The International Union for Conservation of Nature (IUCN) conservation status of these birds is “Least Concerned” with the exception of the albatross which is considered “Near Threatened” [13]. Eggs from these species were sampled over the course of 11 years from 1999–2010, though no data is available from 2007. A majority of the measurements come from the two murre species, and in general, most originate from Alaska. The Laysan albatross is the only class whose samples originate from Hawaii.

This represents a total of 487 unique samples for which 1–3 aliquots were drawn and sent for different chemical analyses, leading to a total of 844 unique aliquots. In total, these aliquots were analyzed for seven different classes of analytes including: (poly)brominated diphenyl ethers (BDEs), variants of (mono-, di-, tri-) butyltin, mercury, organochlorine pesticides (OCPs), polychlorinated biphenyls (PCBs), stable isotope ratios (of carbon and nitrogen), and per- and polyfuoroalkyl substances (PFAS). A total of 174 different analytes were measured across the entire dataset.

Due to co-elution it was not always possible to measure each analyte individually with chromatographic methods, but rather as a pair or triplet. The raw data included a number of such combined sets including: 2,4’ DDT and 4,4’ DDD, BDEs 97 and 118, BDEs 173 and 190, BDEs 204 and 197, PCBs 28 and 31, PCBs 95 and 121, PCBs 106 and 118, PCBs 132 and 153, PCBs 156, 171, and 202, and PCBs 180 and 193. In some cases a different analysis was able to measure individual analytes that in other cases were part of this sum. To standardize this, we combined instances where individual measurements were made to produce the sum, when possible. This is related to our scheme to impute values below the detection limit of instruments. Rather than impute a non-detected value to 0, we generally set this to a random value between zero and the detection limit. If a sum had to be constructed and all contributing analytes fell below their detection limits, the value was taken to be a sum of these random values. By convention, if at least one analyte was detected, the sum was reported as the sum of those detected with no random contribution from non-detected members. This merging reduced the feature space from 174 analytes to 159. This data processing procedure is illustrated in Ref. [15].

These 159 features were further reduced since we required that an analyte (or set thereof) had to be tested for at least 95 per cent of the time (out of the original 487 samples); this does not imply detection, only that a test was performed so the value is not missing. This reduced the number of features to 49. Of the 487 samples, 12 were found to be missing the majority of their measurements and were removed. Moreover, the dataset also contained 14 entries of “Unknown murre” samples; these were also dropped. Thus, the final dataset is a matrix of 461 samples, each with 49 analytes (or sets thereof). Conveniently, this final set contained no missing values and so no imputation is required to use it in practice.

### 3.3 Dataset Information

The dataset’s metadata, including features and their units, are described in the associated files found in Ref. [12]. Briefly, these include columns describing common and species names, geographic area, colony name and associated latitude and longitude, collection year, type of analysis, analyte name, measured value, detection limit and status, and convenience fields for concatenation, grouping, and id linkages.

### 3.4 Unsupervised Analysis

**Fig. 2:**
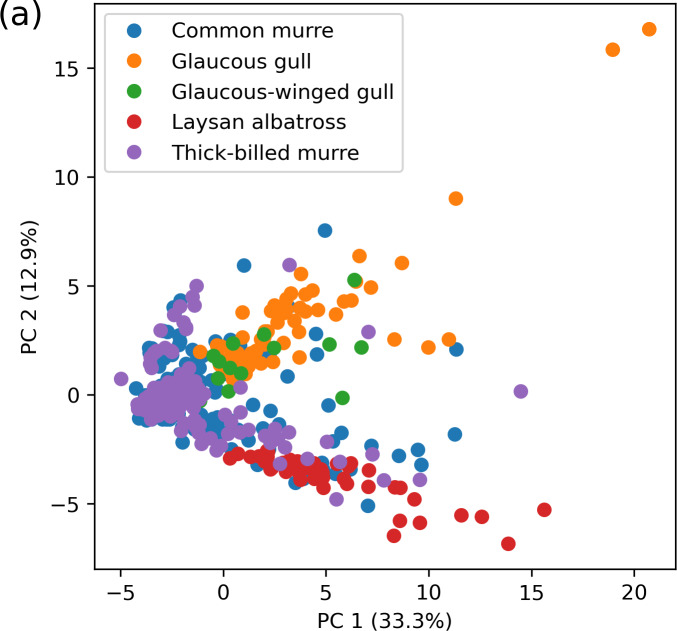

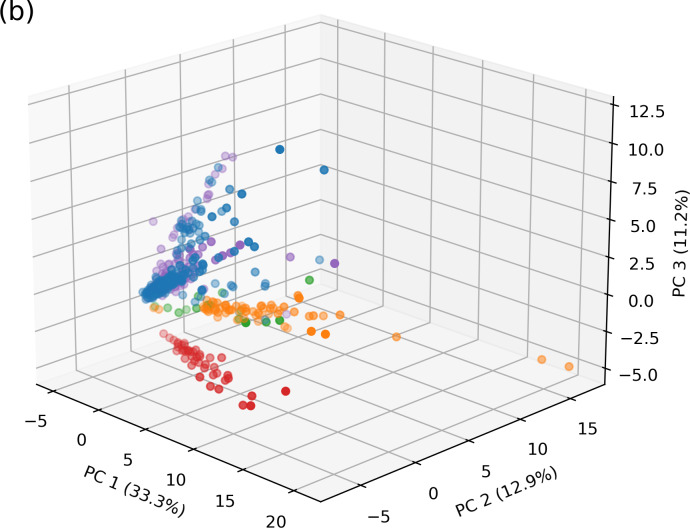

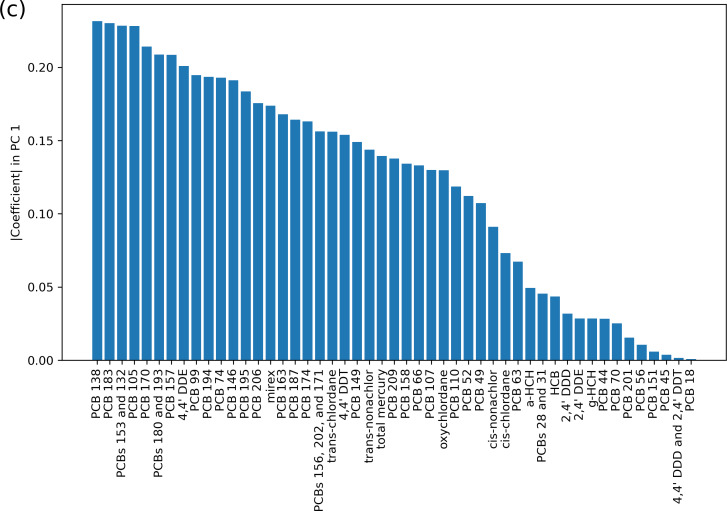
Principal components analysis scores plot of the standardized dataset in terms of the most significant two (a) and three (b) components. Points are colored by species which show some degree of rational separation. The explained variance ratio is expressed as a percentage along each axis. For the leading principal component, the absolute value of the coefficient on each analyte is shown in (c).

To briefly summarize salient characteristics of the dataset we present some unsupervised analysis. First, we performed principal components analysis (PCA) on the standardized dataset. Standardization shifts the mean of column (analyte) to zero and by dividing by its standard deviation, yields a unit variance. Figure 2 illustrates the projection of the data onto the top several components (scores plot). PCA suggests some clustering on the basis of species, though a deeper analysis of this is beyond the scope of this publication.

**Fig. 3:**
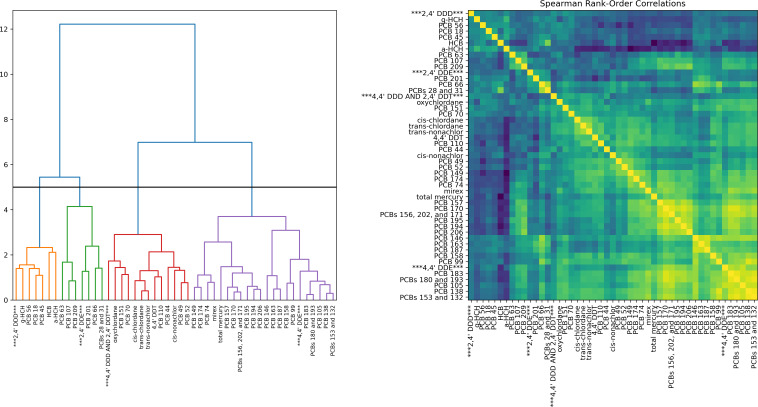
Hierarchical clustering performed on the analytes’ Spearman rank order correlations. Shown at left is the hierarchy from Ward clustering with an arbitrary threshold (black line) that can be used to select the number of clusters. In this example, 4 clusters result and an arbitrarily chosen representative from each is indicated by three asterisks around its name. At right are the Spearman rank-order correlation values themselves for all pairs.

We also consider the degree of correlation that exists between analytes due to similar physical and chemical properties. In addition, many ecological systems’ analytes tend to be correlated; in the case of seabirds, for example, this is due to similarities in the birds’ diet, nesting and breeding locations, and other factors. Figure 3 illustrates hierarchical Ward clustering based on the Spearman rank-order correlation [16] between pairs of analytes after standardization. The correlations and a dendrogram showing the relationship between clusters is depicted. It is possible to select a subset of features by using a threshold to dictate the number of clusters, then select one analyte from each cluster as a representative. The impact of this on models built with different subsets of features is also beyond the scope of this work; we show this merely to illustrate the trends present in the data.

Spearman’s rank order correlation, *r_s_*, is a non-parametric measure of the monotonicity of a pair of analytes, computed by comparing their ordinal ranking [16]; a value of positive or negative 1 corresponds to a perfectly monotonic relationship, while a value of 0 indicates there is no correlation. A larger magnitude indicates that as one analyte increases there is a tendency for the other to increase or decrease, depending on the sign of *r_s_*. Agglomerative clustering using Ward’s linkage [17, 18] can be performed on these rank order correlations to identify analytes that seem to carry similar information, and are therefore redundant. This method of clustering works by combining clusters which lead to the smallest increase in the within-cluster variance following merging [17]. This process is iterated starting from each cluster as a singleton, then sequentially merging until there is only a single cluster. The dendrogram’s vertical axis in Fig. 3 reflects the (Euclidean) distance between cluster centers.

## 4 Impact

As modern computational and data-driven approaches become commonplace in environmental monitoring contexts, the need for robust and well described datasets to validate machine learning models will only increase. This dataset may serve as a canonical reference in the environmental chemometric space or as a component of even larger environmental fate and transport modeling efforts. Individual components of this data set have been used in prior projects, but for the first time are made available in a collated version amenable to machine learning pipelines, which is the subject of future work. This will exemplify the utility of well-curated datasets of this nature.
